# Characterization of the complete plastid genome of *Abies forrestii* (Pinaceae) from southwest China

**DOI:** 10.1080/23802359.2021.1969699

**Published:** 2021-08-25

**Authors:** Na-Lin Dong, Wei Wang, Zhao Wang, Yuan-Yuan Zhang, Yi-Zhen Shao, Hong-Ze Sun

**Affiliations:** a College of Landscape Architecture and Art, Henan Agriculture University, Zhengzhou, China; b College of Life Sciences, Henan Agriculture University, Zhengzhou, China; c Department of Biology, Beijing Dayu School, Beijing, China

**Keywords:** *Abies forrestii*, chloroplast genome, genetic resource, phylogenetic

## Abstract

*Abies forrestii* is endemic to southwest China and ecologically important as a major component of the cold temperate forests. This study was the first report complete chloroplast (cp) genome of *A. forrestii*. The complete chloroplast genome was 120,022 bp in size. In total, 114 genes were identified, including 68 peptide-encoding genes, 35 tRNA genes, four rRNA genes, six open reading frames and one pseudogene. Thirteen genes contain introns. In phylogenetic analysis, *A. forrestii* was found to be closely related with *A. nukiangensis*, *A. fanjingshanensis* and *A. delavayi* subsp. *fansipanensis*. Our study will provide potential genetic resources for further evolutionary studies of this ecologically important species.

The genus *Abies* Miller is the second largest one in the family Pinaceae, consisting of approximately 48 species (Liu 1971). It has a discontinuous distribution in eastern Asia, eastern and western North America, and the Mediterranean basin (extending into Europe and southwest Asia) (Farjon 1990, 2001). *Abies forrestii* Coltm.-Rog. is endemic to southwest China, including northwest of Yunnan province, southwest of Sichuan province and east Tibet. This fir species is ecologically important as a major component of the cold temperate forests and provide a basic home for a great diversity of animals and plants (Liu 1971). Besides, they are also suitable materials for performing conservation, ecological and evolutionary studies (Xiang et al. 2015). Here, we assembled and characterized the complete plastome of *A*. *forrestii*. It will provide potential genetic resources for further relevant evolutionary studies.

The plant material of *Abies forrestii* was collected from a single individual that lives in Daofu county, Sichuan province (30.79°N, 101.29°E). Voucher specimen and DNA sample (Zhang X.-C., No. 5830) were deposited in the herbarium of Institute of Botany, CAS (PE) (http://pe.ibcas.ac.cn/, Qin Ban, herbarium2@ibcas.ac.cn). Total genome DNA was extracted with the Ezup plant genomic DNA prep kit (Sangon Biotech, Shanghai, China). Total DNA was used to generate libraries with an average insert size of 350 bp, which were sequenced using the Illumina HiSeq X platform. In total, ca. 10.2 million high-quality clean reads (150 bp PE read length) were generated with adaptors trimmed. The CLC de novo assembler (CLC Bio, Aarhus, Denmark), BLAST, GeSeq (https://chlorobox.mpimp-golm.mpg.de/geseq.html) (Tillich et al. 2017), and tRNAscan-SE v1.3.1 (Schattner et al. 2005) were used to align, assemble, and annotate the plastome. Genome annotation was performed by comparing the sequences with the cp genomes of *Abies koreana* (KP742350) and *A. neprolepis* (KT834974).

The genome sequence data that support the findings of this study are openly available in GenBank of NCBI at [https://www.ncbi.nlm.nih.gov] under the accession no. MH706715. The associated BioProject, SRA, and Bio-Sample numbers are PRJNA747743, SRP328853 and SAMN20294543 respectively. The full length of *Abies forrestii* chloroplast genome was 120,022 bp. The chloroplast genome showed a typical quadripartite structure that consisted of a pair of IR regions (264 bp) separated by the LSC (66,363 bp) and SSC (53,131 bp) regions, which was similar to the majority of cp genomes in Pinaceae. The GC content were 38.30%. A total of 114 genes were contained in the cp genome (68 peptide-encoding genes, 35 tRNA genes, four rRNA genes, six open reading frames and one pseudogene). Fifty-three protein coding, 16 tRNA genes, three open reading frames and one pseudogene are located in the LSC region, while 15 protein-coding, 17 tRNA genes, 4 rRNA and 3 open reading frames are located in the SSC region, respectively. Only one tRNA gene (trnI-CAU) is duplicated and located on the IR regions. All ndh genes have been lost in the genome of *A*. *forrestii* like other cp genomes of family Pinaceae. Among the protein-coding genes, two genes (rps12 and ycf3) contained two introns, and other eleven genes (trnK-UUU, trnV-UAC, rpoC1, atpF, trnG-GCC, petB, petD, rpl16, rpl2, trnL-UAA, trnA-UGC) had one intron each. In previous studies, short inverted repeat sequences which consist of trnS-psaM-ycf12-trnG and trnG-ycf12-psaM-trnS (1183 bp) are located in 52-kb inversion points of the cp genome of *A*. *forrestii*. Length and sequence of inverted repeats from *A*. *forrestii* is identical with those of *A. koreana* (Yi et al. 2015).

Twenty-one published chloroplast genomes were selected to infer the phylogenetic relationships among 16 different fir species with *Keteleeria davidiana* as the outgroup (Shao et al. 2018, 2019; Fu et al. 2019; Su et al. 2019; Wu et al. 2019; Li et al. 2020; Zhang et al. 2020). These sequences were fully aligned with MAFFT v7.3 (Suita, Osaka, Japan) (Katoh and Standley 2013). For conducting Maximum Likelihood (ML) analyses, the maximum likelihood (ML) inference was performed using GTRþIþC model with RAxML v.8.2.1 (Karlsruhe, Germany) (Stamatakis 2014) on the CIPRES cluster service (Miller et al. 2010). MrBayes was run for 1,000,000 generations, sampling and printing every 100 generations. Based on twenty-two cp genome sequences, these *Abies* species are supported as one monophyletic lineage with extremely high probabilities (BS_ML_ = 100) (Figure 1). And *A. forrestii* was closely related with *A. nukiangensis*, *A. fanjingshanensis* and *A. delavayi* subsp. *fansipanensis* (Figure 1).

**Figure 1.**
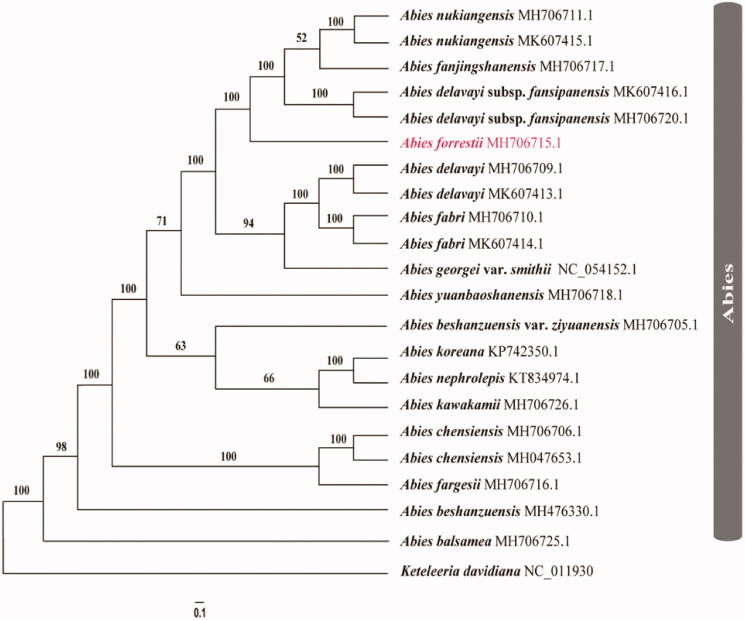
Phylogram of *Abies forrestii* obtained from the maximum likelihood analysis of the whole chloroplast genome sequences. Numbers on branches are support values [maximum likelihood bootstrap values (BS_ML_).

This study provides new insight into the cp genome evolution and phylogenetic relationships of *Abies forrestii*. Moreover, it would be fundamental to formulate potential conservation and management strategies for this ecologically important species of southwest China.

## Data Availability

The genome sequence data that support the findings of this study are openly available in GenBank of NCBI at (https://www.ncbi.nlm.nih.gov/) under the accession no. MH706715. The associated BioProject, SRA, and Bio-Sample numbers are PRJNA747743, SRP328853, and SAMN20294543, respectively.
